# Supplementary material for: Cultural prerequisites of socioeconomic development
Source: R Soc Open Sci. 2020 Feb 12;7(2):190725. doi: 10.1098/rsos.190725 (PMC7062048; doi:10.1098/rsos.190725)
Supplement: Supplementary materials for “Cultural prerequistes of socioeconomic development” [file rsos190725supp1.pdf]

Supplementary materials for "Cultural prerequistes of socioeconomic development"

Damian Ruck
University of Tennessee

Alex Bentley
University of Tennessee

Dan Lawson
University of Bristol

December 7, 2019

Cosmopolitanism and Secular-Rationality

Exploratory Factor Analysis (EFA)

Exploratory Factor Analysis (EFA) is an unsupervised method to uncover any underlying structure in large multivariate dataset by modeling the common variance. EFA assumes each observed variable in the dataset is a weighted linear combination of some set of hidden factors that are to be predicted. This procedure is put more formally in equation S1; where y_n is variable n, F_m is hidden factor m, $w_{n,m}$ is the contribution of factor F_m to variable y_n and ϵ_n is the error term for variable n. We fit this model using maximum likelihood.

$$y_n = w_{n,1}F_1 + w_{n,2}F_2 + ... + w_{n,m}F_m + \epsilon_n \tag{S1}$$

The second part of the EFA regime is an oblique rotation. Rotation acts to improve the interpretability of factors by approaching a simple structure in the factor loading matrix. A factor loading matrix has a perfect simple structure if the following criteria are met:

1. each factor has a small subset of variables with a large factor loadings.
2. the remaining factor loadings should be vanishingly small.
3. each variable should have a large factor loading on one factor only.

The approximate simple structure,obtained using the WEVS data, is illustrated in Figure 1 where each factor is highly loaded by a small set of variables with the bulk having small loadings.

We use an 'oblimin' rotation, an oblique rotation, rather than an orthogonal rotation. An oblique rotation relaxes the orthogonality constraint implicit in EFA, so allows greater freedom when obtaining simple structure. Plus, the

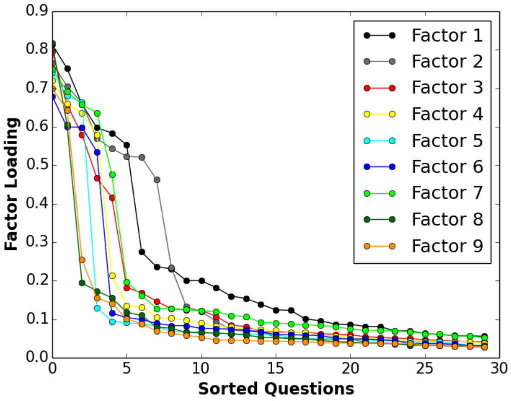

Figure 1: The ordered factor loadings on WEVS survey questions, following EFA analysis with oblique rotation.

orthogonal assumption does not correspond with the real world, where definable cultural values are often correlated.

Given our focus on the interpretability of factors by maximizing simple structure in the factor loading matrix, we chose the number of factors to retain accordingly. The 'Very Simple Structure' (VSS) algorithm selects the number of factors which maximizes simple structure, defined as:

$$\Sigma_{res} = \Sigma - SS^T \tag{S2}$$

$$VSS = 1 - \frac{\Sigma_{res}}{\Sigma} \tag{S3}$$

where Σ is the real correlation matrix of the WEVS data and S is the simplified factor loading matrix. The VSS score can take values between 1 and 0; it takes a value of one when simple structure is present and zero when not. VSS is defined for a given 'complexity' (c), which is the number of factor loadings retained when defining S; for example, if $c = 5$, then all but the highest five factor loadings are set to zero. Σ_{res} is the error created when the simplified correlation matrix SS^T is compared to the real one (Σ). If σ can be recreated using just the c highest factor loadings, then this is the definition of a simple structure and VSS will be close to one.

All aspects of the EFA regime were implemented in R using the 'psych' package.

Nine Cultural Factors

Tables 1-9 contain the significant factor loadings that are used to interpret nine cultural factors —Religiosity, Institutional Confidence, Trust of Norm Violators,

Prosociality, Interest in Politics, Wellbeing, Political Engagement, Respect for Individual Rights, Trust of Out-Groups.

Table 1: **Secularism** - factor 1

WVS question	loading
Important in life: Religion	0.82
How important is God in your life	-0.82
Religious person	0.71
How often do you attend religious services	0.65
Confidence: The Churches	0.64
Important child qualities: Religious faith	0.53

Table 2: **Institutional Confidence** - factor 2

WVS question	loading
Confidence: Parliament	0.81
Confidence: The Government	0.73
Confidence: The Civil service	0.70
Confidence: Political Parties	0.70
Confidence: The police	0.58
Confidence: Labour Unions	0.53
Confidence: Major Companies	0.51
Confidence: The press	0.50
Confidence: The armed forces	0.44

Table 3: **Trust of Norm Violators** - factor 3

WVS question	loading
Neighbors: Drug addicts	0.68
Neighbors: Homosexuals	0.63
Neighbors: Heavy drinkers	0.58
Neighbors: People who have AIDS	0.55

Table 4: **Prosociality** - factor 4

WVS question	loading
Justifiable: Cheating on taxes	0.72
Justifiable: Avoiding a fare on public transport	0.66
Justifiable: Someone accepting a bribe	0.64
Justifiable: Claiming government benefits (not entitled)	0.58

Table 5: **Interest in Politics** - factor 5

WVS question	loading
Important in life: Politics	0.88
Interest in politics	0.59

Table 6: **Wellbeing** - factor 6

WVS question	loading
Satisfaction with your life	-0.71
Feeling of happiness	0.67
State of health (subjective)	0.50
Freedom of choice and control over own life	-0.46

Table 7: **Political Engagement** - factor 7

WVS question	loading
Joining in boycotts	0.74
Attending peaceful demonstrations	0.71
Signing a petition	0.66

Table 8: **Respect for Individual Rights** - factor 8

WVS question	loading
Justifiable: Divorce	0.74
Justifiable: Abortion	0.73
Justifiable: Homosexuality	0.68
Justifiable: Prostitution	0.62
Justifiable: Euthanasia	0.55
Justifiable: Suicide	0.52

Table 9: **Trust of Out-Groups** - factor 9

WVS question	loading
Neighbors: People of a different race	0.72
Neighbors: Immigrants/foreign workers	0.66

Two Cultural Components

The nine cultural factors represent a detailed and interpretable summary of the common variance in the WEVS data. We want to find a more parsimonious summary of the common variance that focuses less on interpretability. Therefore, we ran a weighted PCA using the factor loadings learned by EFA as weights to find a reduced set of orthogonal components underlying the common variance in the entire WEVS data.

Once PCA has been performed, we must decide how many components to retain. The left of figure 2 shows that the first component explains 22% of the variance and the second explains 16% of the variance. The right panel in figure 2 shows us that, of the nine interpretable factors, component one is correlated with Secularism SEC and Political Engagement ENG, whereas component two is correlated with Trust of Norm Violators VIO and Trust of Out-Groups OUT; both are correlated Respect for Individual Rights INV). Therefore we labeled component one 'Secular-Rationality' R and component two as 'Cosmopolitanism' C.

Another reason why we chose two components is that R and C show strong linkages to our measures of a prosperous society (D, E, GDP and L), whereas PC3 and PC4 do not (figure 3).

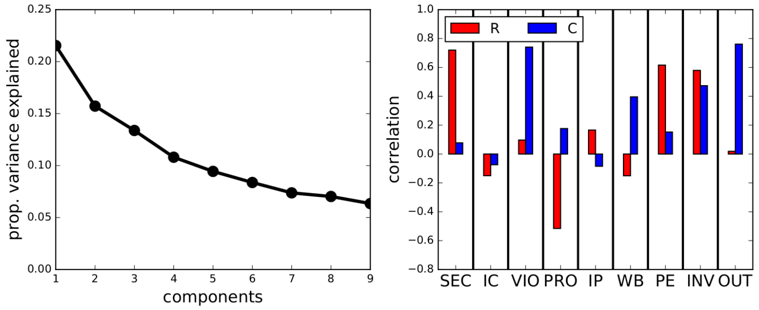

Figure 2: Weighted Principal Component Analysis (PCA) used to compress entire WEVS data into two cultural components (Secular-Rationality R, Cosmopolitanism C). Left: the eigenvalues of the sorted principal components. Top right: the correlations of the nine cultural factors with R and C. Cultural factor abbreviations: SEC = Secularism, IC =Institutional Confidence, VIO = Trust of Norm-Violators, PRO = Prosociality, IP = Interest in Politics, WB = Wellbeing, PE = Political Engagement, INV = Respect for Individual Rights, OUT = Trust in Out-Groups.

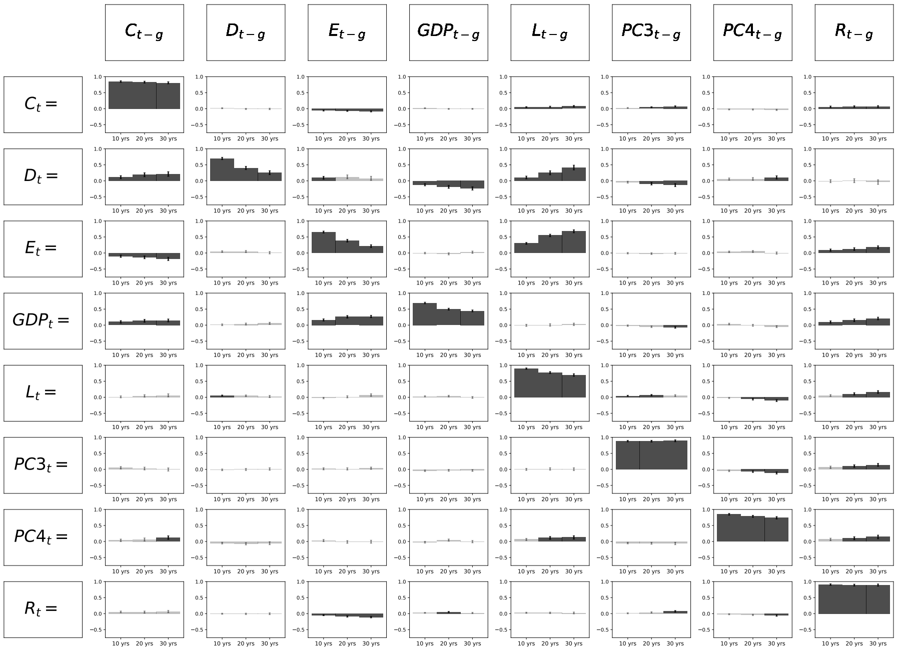

Figure 3: Multi-level time-lagged linear regression results (equation 1 in main text), but including rejected principal components PC3 and PC4. It shows the effect sizes with time lag of g=10, 20 and 20 years. If a 95% credible interval is met then the bars are solid and black error bars are standard errors. Variable abbreviations: D = Democracy, GDP = GDP per capita, E = Education, L = Life Expectancy, R = Secular-Rationality and C = Cosmopolitanism

Why not use PCA on the raw WEVS data?

We used a weighted PCA to derive R and C because using the factor loadings from the EFA as weightings ensures that we created smoothed components with the noise (generated by single questions) removed. Running a PCA directly on the raw WEVS data, means we are parsimoniously expressing the *entire* WEVS variance, including noise. By running PCA directly, we retrieve two noisy representations of C and R with respective Pearson correlation coefficients $r_R = 0.55$ and $r_C = 0.47$. Tables 10 and 11 show the significant factor loadings ($|r| > 0.4$) for the PC's measured directly from the WEVS.

Table 10: PC1 direct - Secular-Rationality

**WEVS question**	**load.**
Important in life: Religion	0.7
How important is God in your life	0.69
Justifiable: abortion	0.64
How often do you attend religious services	0.56
Confidence: Churches	0.56
Justifiable: divorce	0.55
Important child qualities: religious faith	0.53
Religious person	0.53
Justifiable: suicide	0.52
Justifiable: euthanasia	0.51
Justifiable: homosexuality	0.51
Justifiable: prostitution	0.48

Table 11: PC3 direct - Cosmopolitanism

**WEVS question**	**load.**
Neighbours: People who have AIDS	0.53
Neighbours: Homosexuals	0.51
Satisfaction with your life	0.5
Political action: signing a petition	0.47
Feeling of happiness	0.45
Justifiable: homosexuality	0.4

Independence of birth decade from time period

We split each WEVS period p by birth decade t which gives us a tensor for each cultural component $X_{g,p,n}$. We then average across all time periods to get the the birth decade trend X_g for each nation n. However, we get significant variation across WEVS periods, so to avoid biasing the birth decade average, we impute missing birth decade using a linear model on birth decades; table illustrates the values that are imputed. We also impute using a quadratic function to ensure linear imputation does not unduly affect the results.

t\p	1990-1995	1995-2000	2000-2005	2005-2010	2010-2015
**1900**					
**1910**	-0.292	-0.379		impute	impute
**1920**	-0.286	-0.302		-0.199	impute
**1930**	-0.112	-0.105		-0.145	-0.076
**1940**	0.00	-0.01		0.069	0.041
**1950**	0.033	0.098		0.248	0.138
**1960**	0.02	-0.013		0.158	0.136
**1970**	0.076	0.124		0.095	0.147
**1980**	impute	0.188		0.215	0.275
**1990**	impute	impute		impute	0.339

Table 12: Example period-birth decade (p-g) matrices for Secular-Rationality R in the USA. The rows are generations and the columns are time periods and each entry is the mean R of the sample for that WEVS period and birth decade combination. The entries labeled "impute" are the values we impute to avoid biases in birth decade averages.

For a given nation n, the birth decade time series for arbitrary cultural value X_g seems to be stable across time periods p (figure 4a). However, to test if this is generally true, we run a series of hierarchical regressions which explain $X_{g,p,n}$ in terms of continuous birth cohort g and discrete time period p and nation n.

We compare the out-of-sample explanatory power of four regressions which increase in complexity. The basic model being:

$$X_{g,p,n} = \alpha + \beta g + \epsilon$$

where α and β are hierarchical intercept and slope parameters respectively; g is birth decade and ϵ is error.

The hierarchical parameter structure of the four models are as follows:

$$M1 : \alpha = \alpha_0 + \alpha_n, \quad \beta = \beta_0 + \beta_n$$

$$M2.1 : \alpha = \alpha_0 + \alpha_n, \quad \beta = \beta_0 + \beta_{n,p}$$

$$M2.2 : \alpha = \alpha_0 + \alpha_{n,p}, \quad \beta = \beta_0 + \beta_n$$

$$M3 : \alpha = \alpha_0 + \alpha_{n,p}, \quad \beta = \beta_0 + \beta_{n,p}$$

where M1 assumes a unique intercept and slope for each nation. M2.1 adds complexity by assuming a unique slope for each period and nation combination and M2.2 assumes a unique intercept (rather than a unique slope). Finally, M3 is the most complex because it assumes both a unique intercept and slope for each period and nation combination.

We calculate the out-of-sample predictive capacity of the four models using the Akaike Information Criterion (AIC), Deviance Information Criterion (DIC) and Widely Applicable Information Criterion (WAIC); these all penalize overfitting using the effective number number of parameters. The lower the information criterion, the better the model performs.

We treat time period p as a discrete random effect, unlike birth decade g, which we treat as continuous and linear. This is because period changes are rapid and transient, unlike birth decade changes which are gradual and steady.

Figure 4b confirms our hypothesis that birth decade time series are approximately stable with time period ($X_{g,n} \approx X_{g,p,n}$). Adding a unique period-nation intercept ($M2.2$) markedly improves the AIC compared to both the simplest model ($M1$) and the unique period-nation slope model ($M2.1$). Crucially, however, the information criteria do not significantly decrease for the model with a unique period-nation intercept and slope ($M3$).

This demonstrates that birth decade time series are effectively independent of time period and is why, when we run our hierarchical time-lagged linear regressions using birth decade time series from specific time periods (p=1990,1995,2000,2005,2010), the results are qualitatively the same (see Sensitivity Analysis).

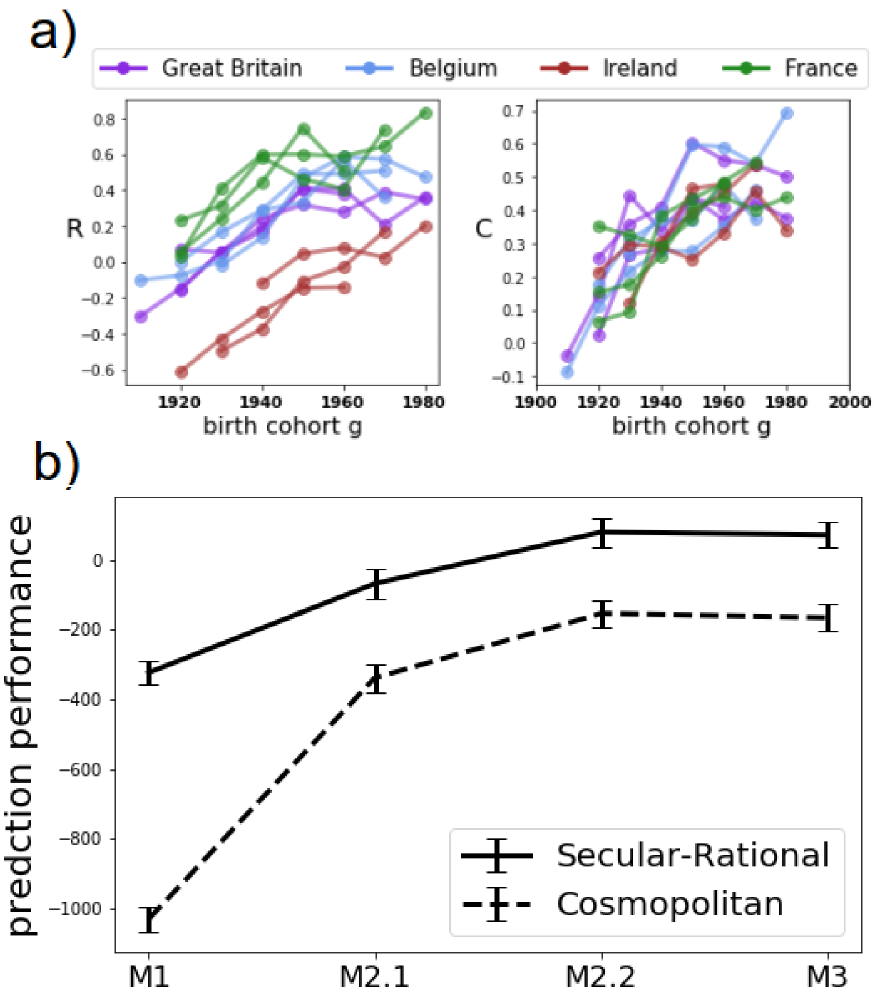

Figure 4: The stability of birth decade differences in each time period for all four cultural values (Secular-Rationality R and Cosmopolitanism C): a) birth decade time series for each time period in four example nations; b) two-fold cross validation results (performance assessed using 'expected log pointwise predictive density') comparing hierarchical models of increasing complexity that explain cultural value change in terms of continuous birth decade g and categories for time period p and nation n; where M1 is the simplest model (unique intercept and slope for each nation) and M3 is the most complex (unique intercept and slope for each period-nation combination).

Simulation study: Do birth decades represent past time periods?

Underpinning our analysis is the assumption that birth decades can be used to represent the cultural values of past time periods. Here we run simulations

to show that, even in the presence of significant period effects — with a cycle length of up to 60 years — birth years are predictive of past time periods.

First, we assume successive yearly birth cohorts g that are increasing in some arbitrary cultural value X. We assume this is at first a linear change $X_g = normal(300g, 200)$ and then a quadratic change $X_g = normal(-g^2+g, 200)$. We tuned these functions so their total displacements are roughly in proportion.

Cultural values at each time period, X_t, is the average of all the adult birth years (aged between 20 and 69), plus a cyclical independent period effect:

$$X_t = \frac{1}{50}\sum_{h=20}^{69} X_{t,(g=t-h)} + 1000sin(\omega t)$$

where, t is time period, h is age and ω is the speed of the period change (2, 5, 10, 20, 40 or 60 years). We simulate three nations for each case (see figure 5a for example time series).

Using the simulated data, we test if X_g can be used to predict X_t, a) in the presence of large period effects and b) when we do not know the age when a birth year becomes active in the population (age 20 years in the simulation). We use the following regression:

$$X_{t,n} = \alpha + \alpha_n + \beta X_{g,n} + \epsilon \tag{1}$$

where, α is global intercept, α_n is nation intercept and β is the coefficient of proportionality between birth decade and cross-sectional time series.

Figure 5b shows that birth decades are representative of past time periods in the presence of both period change (any cycle rate from 2 to 60 years) and uncertainty surrounding the age of adulthood (we assume the age is 0-10, 10-20 and 20-30 years). Increasing the speed of period change seems to reduce the detected effect size comapred to that detected with zero period change.

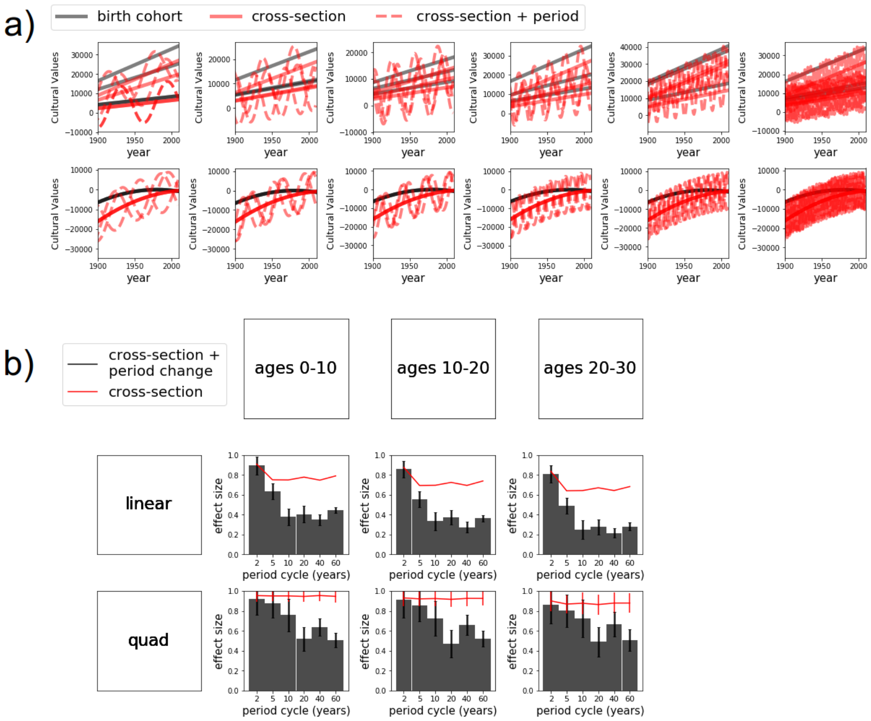

Figure 5: Simulation of cultural value change driven by both birth decade replacement and period change. a) simulated cultural value time series with linear (top row) and quadratic (bottom row) birth decade change superpositioned with period change cycles of 60, 40, 20, 10, 5, 2 years (columns). b) effect size of simulated birth decade time series on cross-sectional time series, with no period change (red line) and period change (black bars)

Language family

Language category assignments for the 109 WEVS nations.

Table 13: The language category assigned to each country from the WEVS

**country**	***language***	**country**	***language***	**country**	***language***
Albania	*Albania*	Greece	*Greek-Armenian*	Peru	*Italic*
Algeria	*Semitic*	Guatemala	*Italic*	Philippines	*Austronesian*
Andorra	*Italic*	Hong Kong	*Sino-Tibetan*	Poland	*Balto-Slavic*
Argentina	*Italic*	Hungary	*Uralic*	Portugal	*Italic*
Armenia	*Greek-Armenian*	Iceland	*Germanic*	Puerto Rico	*Italic*
Australia	*Germanic*	India	*Indo-Aryan*	Qatar	*Semitic*
Austria	*Germanic*	Indonesia	*Austronesian*	Romania	*Italic*
Azerbaijan	*Turkic*	Iran	*Indo-Aryan*	Russia	*Balto-Slavic*
Bahrain	*Semitic*	Iraq	*Semitic*	Rwanda	*Niger_Congo*
Bangladesh	*Indo-Aryan*	Ireland	*Germanic*	Saudi Arabia	*Semitic*
Belarus	*Balto-Slavic*	Israel	*Semitic*	Serbia	*Balto-Slavic*
Belgium	*Germanic*	Italy	*Italic*	Singapore	*Germanic*
Bosnia	*Balto-Slavic*	Japan	*Japan*	Slovakia	*Balto-Slavic*
Brazil	*Italic*	Jordan	*Semitic*	Slovenia	*Balto-Slavic*
Bulgaria	*Balto-Slavic*	Kazakhstan	*Turkic*	South Africa	*Germanic*
Burkina Faso	*Italic*	Kosovo	*Albania*	South Korea	*Korea*
Canada	*Germanic*	Kuwait	*Semitic*	Spain	*Italic*
Chile	*Italic*	Kyrgyzstan	*Turkic*	Sweden	*Germanic*
China	*Sino-Tibetan*	Latvia	*Balto-Slavic*	Switzerland	*Germanic*
Colombia	*Italic*	Lebanon	*Semitic*	Taiwan	*Sino-Tibetan*
Croatia	*Balto-Slavic*	Libya	*Semitic*	Tanzania	*Niger_Congo*
Cyprus (G)	*Greek-Armenian*	Lithuania	*Balto-Slavic*	Thailand	*Tai*
Cyprus (T)	*Turkic*	Luxembourg	*Italic*	Trinidad	*Germanic*
Czech Rep.	*Balto-Slavic*	Macedonia	*Balto-Slavic*	Tunisia	*Semitic*
Denmark	*Germanic*	Malaysia	*Austronesian*	Turkey	*Turkic*
Dominican Rep.	*Italic*	Mali	*Italic*	Uganda	*Niger_Congo*
Ecuador	*Italic*	Malta	*Semitic*	Ukraine	*Balto-Slavic*
Egypt	*Semitic*	Mexico	*Italic*	United States	*Germanic*
El Salvador	*Italic*	Moldova	*Italic*	Uruguay	*Italic*
Estonia	*Uralic*	Morocco	*Semitic*	Uzbekistan	*Turkic*
Ethiopia	*Semitic*	Netherlands	*Germanic*	Venezuela	*Italic*
Finland	*Uralic*	New Zealand	*Germanic*	Viet Nam	*Austroasiatic*
France	*Italic*	Nigeria	*Germanic*	Yemen	*Semitic*
Georgia	*Kartvelian*	North Ireland	*Germanic*	Zambia	*Germanic*
Germany	*Germanic*	Norway	*Germanic*	Zimbabwe	*Germanic*
Ghana	*Germanic*	Pakistan	*Indo-Aryan*		
Great Britain	*Germanic*	Palestine	*Semitic*		

Sensitivity Analysis

Age of Adulthood

In the main text, we assume that a birth decade first begins to influence society between the ages of 10-20 years. However, we recognize that this an assumption that could have consequences, given that we are essentially representing past time periods using youngest birth decades. Nonetheless, when we re-run our analysis assuming the age of adulthood is between 0-10 (figure 6) and 20-30 (figure 7), our main findings remain the same.

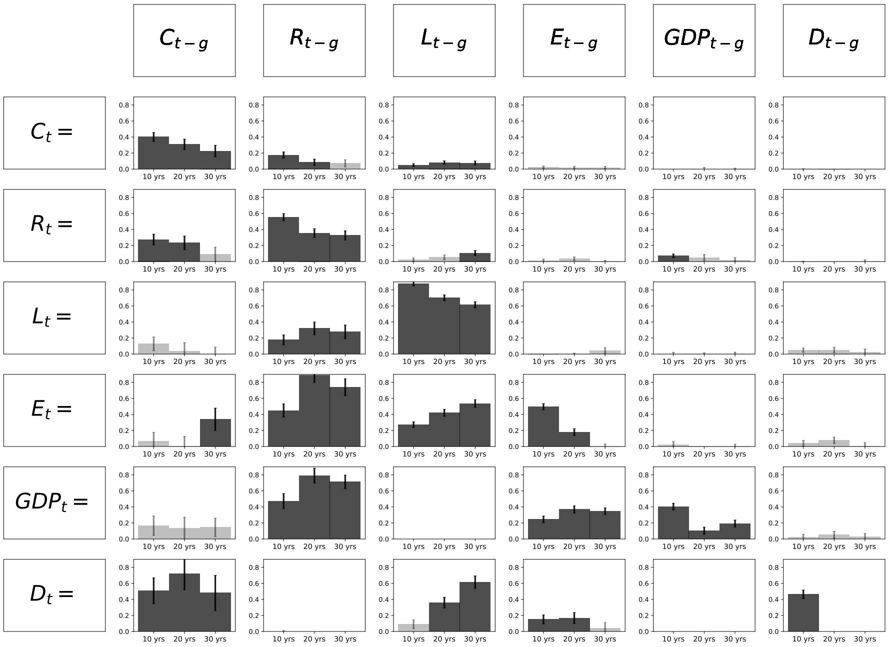

Figure 6: Results from the multi-level time-lagged linear regression assuming birth decades first affect society between the ages 0-10 years. It shows the effect sizes with time lag of g=10, 20 and 20 years. If a 95% credible interval is met then the bars are solid and black error bars are standard errors. Variable abbreviations: D = Democracy, GDP = GDP per capita, E = Education, L = Life Expectancy, R = Secular-Rationality and C = Cosmopolitanism

Quadratic birth decade imputation

We determined birth decade time series by averaging over all time periods. However, because period changes can be large, we had to impute birth decades that were missing from certain time periods. In the main text we did this using

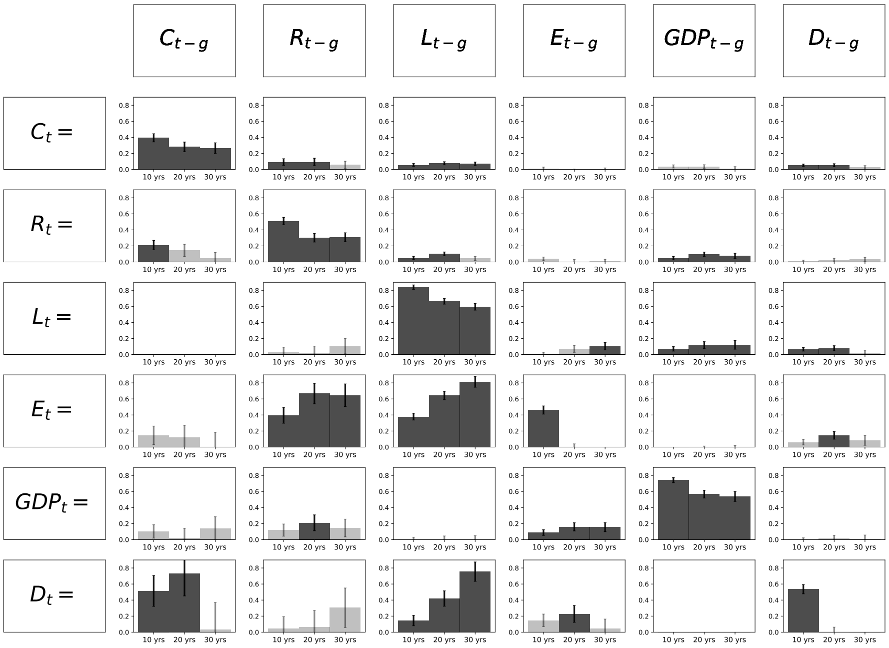

Figure 7: Results from the multi-level time-lagged linear regression assuming birth decades first affect society between the ages 20-30 years. It shows the effect sizes with time lag of g=10, 20 and 20 years. If a 95% credible interval is met then the bars are solid and black error bars are standard errors. Variable abbreviations: D = Democracy, GDP = GDP per capita, E = Education, L = Life Expectancy, R = Secular-Rationality and C = Cosmopolitanism

a linear model. Here we do so using a quadratic model and show that our main findings are preserved.

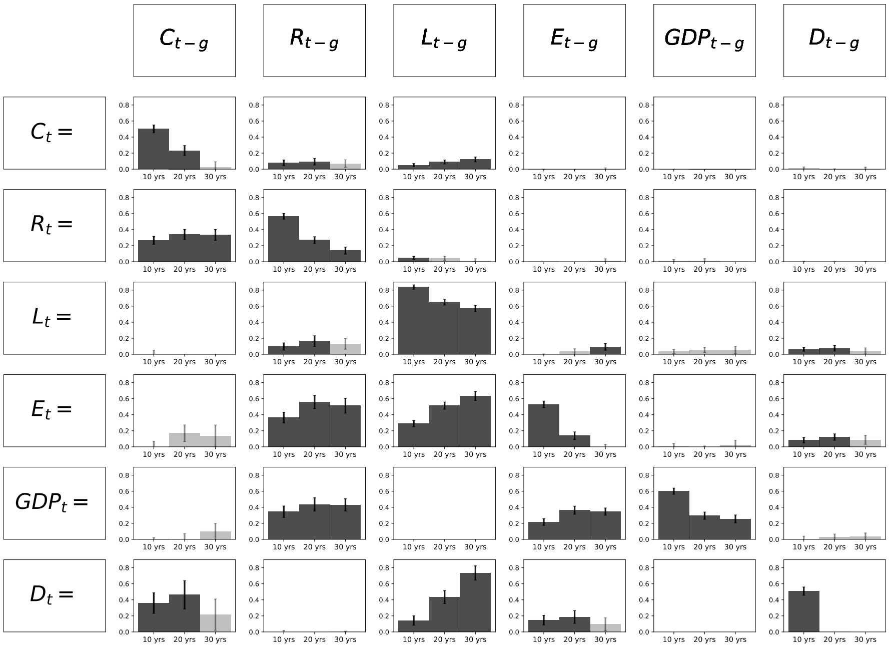

Figure 8: Results from the multi-level time-lagged linear regression, but imputing missing birth decades using a quadratic model. It shows the effect sizes with time lag of g=10, 20 and 20 years. If a 95% credible interval is met then the bars are solid and black error bars are standard errors. Variable abbreviations: D = Democracy, GDP = GDP per capita, E = Education, L = Life Expectancy, R = Secular-Rationality and C = Cosmopolitanism

Birth decade time series from single time periods

We are able to average birth decade time series over all time periods because, as shown in a previous section, birth decade time series are independent of time period. Therefore, we should expect to attain the same regression results irrespective of the time period when the birth decade time series were measured. As figure 9) shows, the results are qualitatively the same regardless of the time period used.

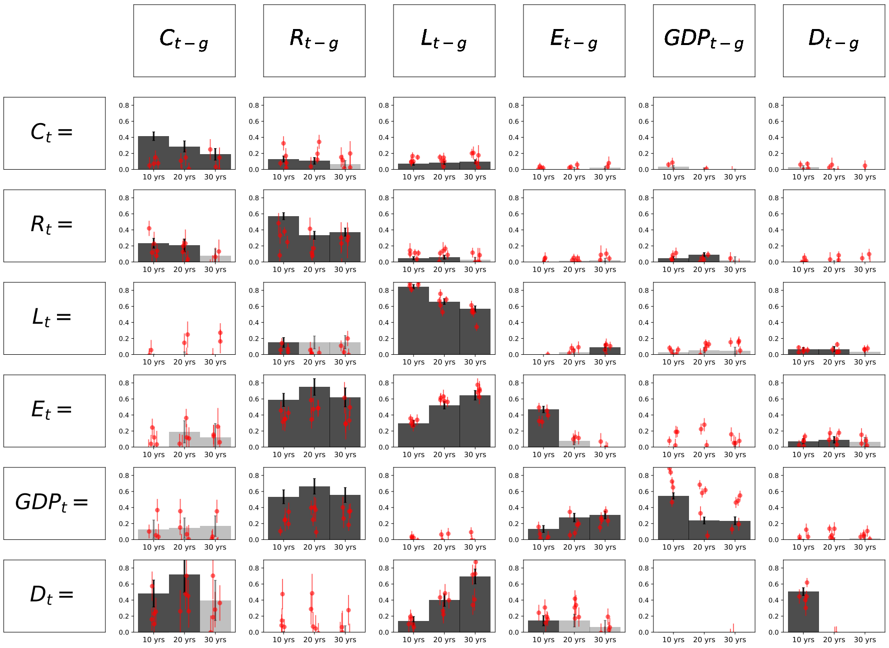

Figure 9: Results from the multi-level time-lagged linear regression for both birth decade time series R and C averaged over all time periods (black bars) and for each individual time period (red bars). If a 95% credible interval is met then the bars are solid and black error bars are standard errors. Variable abbreviations: D = Democracy, GDP = GDP per capita, E = Education, L = Life Expectancy, R = Secular-Rationality and C = Cosmopolitanism

Substitute highly correlated cultural factors

Derived in previous sections, Secular-Rationality R and and Cosmopolitanism C are orthogonal principal components that efficiently represent the WEVS data. To show that these orthogonal summaries are meaningful, we substitute R and C with the interpretable cultural factors (see previous section) that are highly correlated with them (R with secularism SEC, political engagement ENG and respect for individual rights and I; C with trust of out-groups OUT, trust of norm-violators VIO and respect for individual rights I).

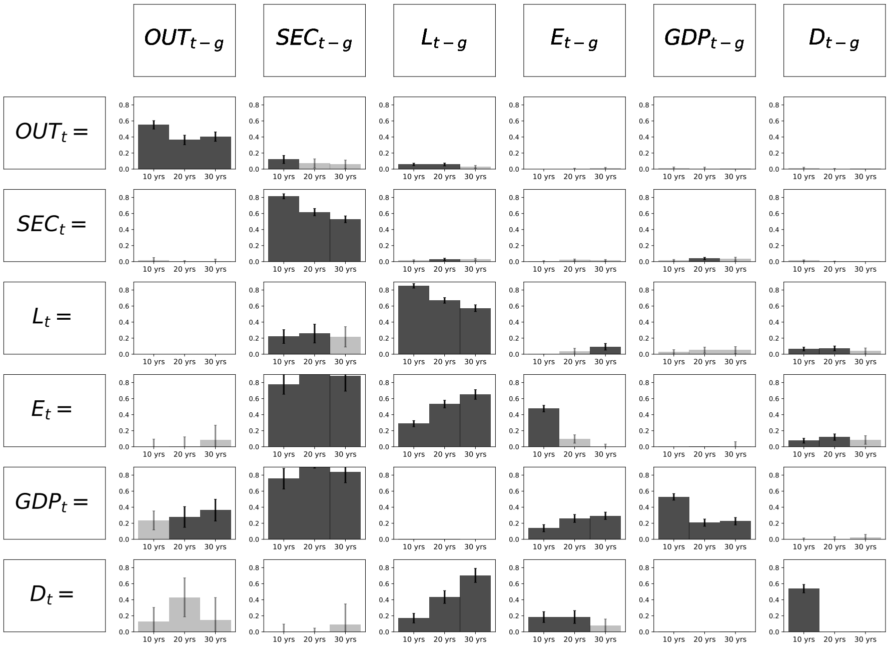

Figure 10: Comparable regression results to those presented in figure 1, but $R = SEC$ and $C = OUT$. It shows the effect sizes with time lag of g=10, 20 and 20 years. If a 95% credible interval is met then the bars are solid and black error bars are standard errors. Variable abbreviations: D = Democracy, GDP = GDP per capita, E = Education, L = Life Expectancy

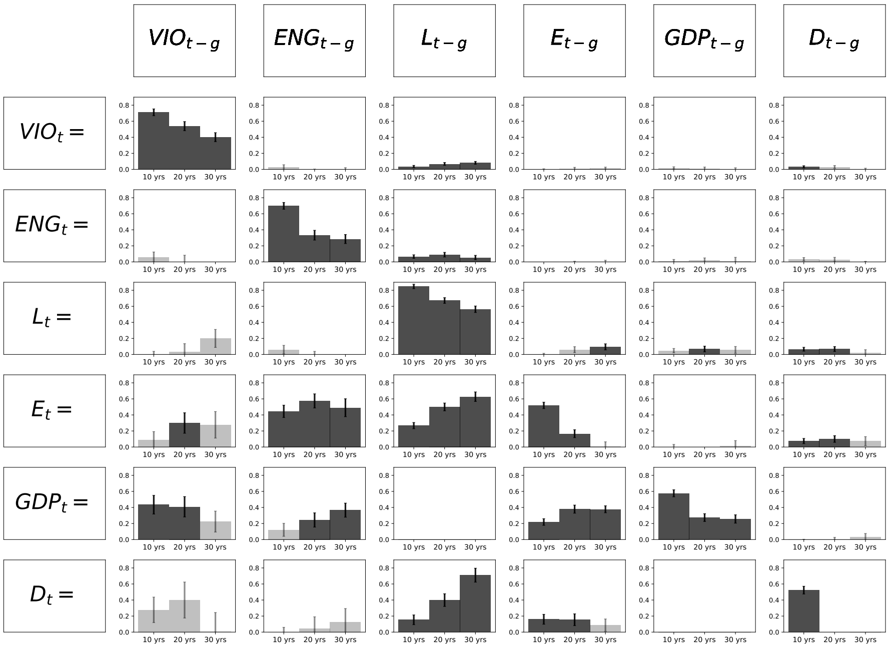

Figure 11: Comparable regression results to those presented in figure 1, but $R = ENG$ and $C = VIO$. It shows the effect sizes with time lag of g=10, 20 and 20 years. If a 95% credible interval is met then the bars are solid and black error bars are standard errors. Variable abbreviations: D = Democracy, GDP = GDP per capita, E = Education, L = Life Expectancy

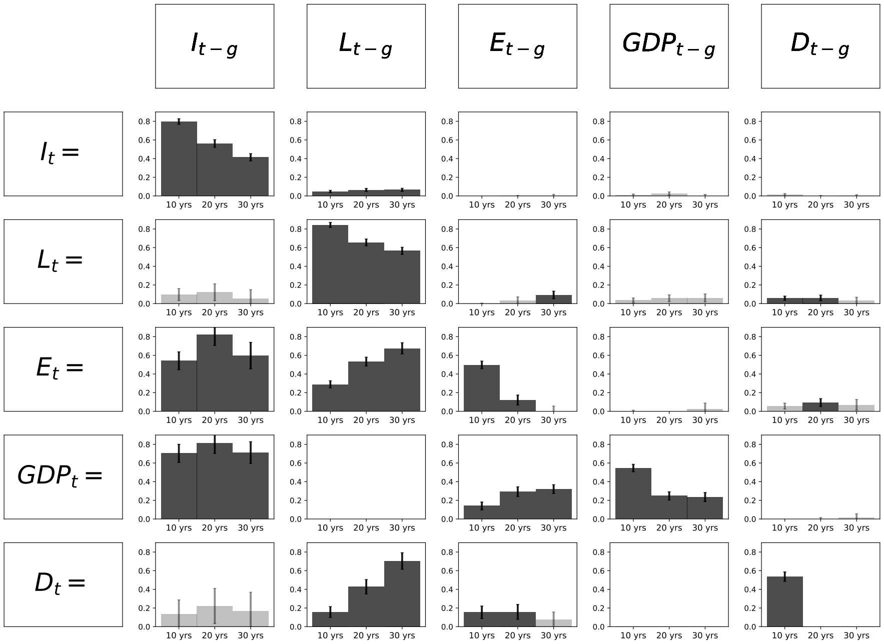

Figure 12: Comparable regression results to those presented in figure 1, but $R = C = I$. It shows the effect sizes with time lag of g=10, 20 and 20 years, and for each lag we assume a birth decade becomes active during at 0-10 years. If a 95% credible interval is met then the bars are solid and black error bars are standard errors. Variable abbreviations: D = Democracy, GDP = GDP per capita, E = Education, L = Life Expectancy

Full regression tables

In the main text, we have graphically represented the lagged fixed effects of R, C, GDP, D, E and L on each other, including standard errors and credible intervals. Here we report tables containing the full results; including summaries (mean, standard error, credible intervals) and diagnostic statistics (effective sample size n_{eff} and convergence R_{hat}) for all parameters in the hierarchical joint posterior distribution. These results are for a time lag of 2 decades and assume the age of adulthood is between 10 and 20 years.

Dependent variable: Cosmopolitanism C

	Mean	StdDev	lower 0.89	upper 0.89	n_eff	Rhat
alpha_g	-0.351525	3.028324	-5.037549	4.520611	1800.138764	0.998747
alpha_c[1]	0.165711	3.205934	-4.708045	5.376597	814.075341	1.004517
alpha_c[2]	0.496554	2.300136	-3.103666	4.182348	445.685105	1.002618
alpha_c[3]	0.265576	2.391985	-3.581891	4.057656	501.932494	1.003129
alpha_c[4]	0.140819	2.392850	-3.793420	3.833465	504.499740	1.003139
alpha_c[5]	-0.069909	4.250701	-7.222529	6.344529	1446.330932	1.000427
alpha_c[6]	0.169968	2.392393	-3.728382	3.895441	502.734009	1.003182
alpha_c[7]	0.215655	2.301507	-3.365385	3.943434	449.964054	1.002613
alpha_c[8]	-0.167700	4.133095	-6.208237	6.546927	1445.333641	1.001529
alpha_c[9]	0.366705	2.392813	-3.479306	4.156659	503.041012	1.003201
alpha_c[10]	-0.018572	2.299185	-3.495940	3.809792	447.573141	1.002658
alpha_c[11]	-0.500114	6.036486	-10.848751	8.705678	2467.451828	0.999354
alpha_c[12]	0.131071	2.299467	-3.325310	3.989006	451.454291	1.002606
alpha_c[13]	-0.134343	5.799705	-9.758476	8.464130	2161.297678	1.000619
alpha_c[14]	0.380737	4.132227	-5.800720	7.019430	1451.431676	1.001416
alpha_c[15]	0.504570	2.396293	-3.319642	4.328152	501.810199	1.003266
alpha_c[16]	-0.421600	2.303604	-3.860527	3.447845	453.958209	1.002734
alpha_c[17]	0.174424	3.210700	-4.657350	5.473110	815.749282	1.004584
alpha_c[18]	-0.581278	2.303934	-4.094094	3.255546	449.588646	1.002744
alpha_c[19]	0.312117	5.741863	-9.132065	9.117628	2379.972111	0.999705
alpha_c[20]	0.140474	2.300500	-3.247900	4.059811	451.273805	1.002466
alpha_c[21]	-0.036717	2.393296	-3.952155	3.724991	503.585295	1.003090
alpha_c[22]	-0.884111	2.392630	-4.699321	2.936727	503.343306	1.003254
alpha_c[23]	0.225122	2.392049	-3.359030	4.305976	501.609868	1.003076
alpha_c[24]	0.377411	5.802371	-9.181632	9.089856	2168.662850	1.000646
alpha_c[25]	0.276635	2.300468	-3.232951	4.080023	452.540976	1.002525
alpha_c[26]	-0.361854	5.741563	-9.945668	8.316772	2374.243615	0.999711
alpha_c[27]	-0.318551	4.254424	-6.762820	6.851426	1445.852567	1.000457
alpha_c[28]	-0.140789	5.239051	-8.333942	8.138782	2136.108638	1.000738
alpha_c[29]	0.142499	4.252747	-6.957877	6.633936	1437.989132	1.000406
alpha_c[30]	0.244042	3.208384	-4.643822	5.457495	815.133946	1.004502
alpha_c[31]	0.312755	2.392643	-3.529623	4.107275	503.110074	1.003123
alpha_c[32]	0.045703	2.300442	-3.434764	3.879543	451.810396	1.002560
alpha_c[33]	0.200742	7.242105	-10.362911	12.669101	2814.741081	1.000975
alpha_c[34]	-0.103095	3.204623	-4.777633	5.323214	812.669549	1.004609
alpha_c[35]	0.132948	3.204768	-4.701586	5.453040	815.936491	1.004465
alpha_c[36]	-0.263132	3.205681	-5.179983	4.917237	815.481400	1.004530
alpha_c[37]	0.136006	2.300342	-3.607094	3.737595	447.879556	1.002486
alpha_c[38]	-0.367289	5.240749	-8.689841	7.768118	2139.449879	1.000733
alpha_c[39]	-0.861611	2.305964	-4.656359	2.652998	451.008983	1.002795
alpha_c[40]	-0.169290	2.298707	-3.956692	3.344330	450.655004	1.002585
alpha_c[41]	0.486915	3.210118	-4.336586	5.792873	814.772422	1.004551
alpha_c[42]	0.361674	2.394026	-3.447946	4.195854	503.610110	1.003193
alpha_c[43]	0.231589	2.393761	-3.628610	3.997308	501.795030	1.003159
alpha_c[44]	-0.100710	4.639829	-7.795377	7.115594	1581.355060	0.999840
alpha_c[45]	0.495398	2.394156	-3.042390	4.622884	501.329937	1.003123
alpha_c[46]	0.680921	4.254033	-6.312211	7.322127	1433.018333	1.000401
alpha_c[47]	-0.268177	2.301341	-3.933648	3.379802	450.056883	1.002716
alpha_c[48]	-0.028094	5.238069	-8.123225	8.334155	2144.052218	1.000666
alpha_c[49]	0.441819	4.131121	-5.821222	6.956457	1440.107919	1.001549

	Mean	StdDev	lower 0.89	upper 0.89	n_eff	Rhat
alpha_c[50]	0.051996	2.300364	-3.375235	3.903617	451.748295	1.002659
alpha_c[51]	-0.802041	2.308858	-4.608737	2.686899	451.358518	1.002663
alpha_c[52]	-0.499137	4.129973	-6.801219	6.005256	1445.582836	1.001445
alpha_c[53]	0.055556	4.130462	-6.005389	6.738895	1439.484494	1.001545
alpha_c[54]	-0.633507	2.389349	-4.466606	3.165496	504.491577	1.003157
alpha_c[55]	-0.027366	7.323852	-12.790142	10.745043	2591.603037	0.999280
alpha_c[56]	0.329235	2.301522	-3.131634	4.183314	444.851033	1.002594
alpha_c[57]	0.387962	2.392193	-3.023951	4.627145	502.633830	1.003152
alpha_c[58]	0.431720	2.391631	-3.185845	4.485788	504.160340	1.003138
alpha_c[59]	0.377142	6.035810	-9.920671	9.588002	2470.224889	0.999346
alpha_c[60]	-0.060064	7.059652	-10.767944	11.254734	3383.232196	0.999764
alpha_c[61]	-0.124210	2.393022	-3.932582	3.692139	503.758243	1.003174
alpha_c[62]	0.679361	3.207780	-4.167639	5.922740	813.914450	1.004627
alpha_c[63]	-0.172682	7.155541	-11.577508	11.214960	2751.740629	0.998932
alpha_c[64]	-0.127227	4.639065	-6.841853	7.973961	1569.831844	0.999856
alpha_c[65]	0.039619	2.391303	-3.771308	3.878140	501.331596	1.003224
alpha_c[66]	0.453231	2.300600	-3.079226	4.236718	448.108574	1.002596
alpha_c[67]	-0.391931	2.300947	-3.815593	3.504804	451.319553	1.002555
alpha_c[68]	-0.119493	3.209483	-4.981577	5.103374	815.588480	1.004576
alpha_c[69]	-0.153130	4.637476	-6.660977	8.211201	1580.872846	0.999833
alpha_c[70]	0.158592	4.643199	-6.497634	8.335234	1581.821979	0.999839
alpha_h[1]	-0.703476	4.211024	-7.469644	6.067473	1211.637451	1.000755
alpha_h[2]	0.708469	3.722101	-5.005919	6.782189	1095.740517	0.999005
alpha_h[3]	0.665269	3.673031	-5.058925	6.449735	919.253751	1.000716
alpha_h[4]	-0.275803	4.896703	-8.240099	7.329995	1529.606208	1.000119
alpha_h[5]	-0.145318	4.894039	-7.993672	7.580741	1514.656500	1.001038
alpha_h[6]	-0.060203	6.099510	-9.477333	10.141101	2542.427065	0.999257
alpha_h[7]	0.309341	6.190580	-9.321087	10.036832	2008.917956	1.000762
alpha_h[8]	0.327695	5.970684	-9.070049	9.296669	2755.712205	0.999651
alpha_h[9]	-0.241739	5.543153	-8.387618	9.180850	2209.104086	1.000590
alpha_h[10]	-0.092546	7.181981	-12.314220	10.835397	2819.222530	1.000767
alpha_h[11]	-0.265437	5.201539	-8.343738	8.333250	1725.678607	0.999908
alpha_h[12]	-0.389849	7.204397	-12.395961	10.795623	2603.207631	0.999380
alpha_h[13]	-0.351998	7.218135	-11.774732	10.555701	3226.769569	0.999210
alpha_h[14]	-0.346076	7.108443	-11.093643	11.429460	3098.338306	0.998611
sigma	0.125167	0.006268	0.115058	0.134828	1129.171967	1.002319
betaGDP	-0.001000	0.022206	-0.035353	0.034302	2858.385737	0.998578
betaRAT	0.106723	0.041923	0.042693	0.176837	2824.737281	0.998629
betaCOS	0.287255	0.069079	0.174331	0.399559	3322.065787	0.998501
betaEDS	0.005597	0.018974	-0.023489	0.035286	2843.186378	0.999132
betaLEX	0.081738	0.020523	0.049521	0.113771	2727.735412	0.998478
betaDEM	-0.010356	0.020992	-0.042408	0.025467	2499.783301	1.000173

Dependent variable: Secular-Rationalism R

	Mean	StdDev	lower 0.89	upper 0.89	n_eff	Rhat
alpha_g	0.055884	3.056150	-4.869158	4.935653	1600.453675	1.003254
alpha_c[1]	0.202842	3.246026	-4.745986	5.620254	812.526328	1.000991
alpha_c[2]	-0.246376	2.297889	-3.910104	3.356346	444.973857	1.001086
alpha_c[3]	0.220208	2.373673	-3.817058	3.839360	555.097169	1.001781
alpha_c[4]	0.328825	2.371917	-3.444931	4.191796	556.863421	1.001786
alpha_c[5]	-0.269731	4.471629	-7.276100	7.071879	1530.937064	1.000887
alpha_c[6]	0.325749	2.374672	-3.545063	4.096553	552.816021	1.001782
alpha_c[7]	-0.230289	2.298941	-3.836939	3.399018	446.075002	1.001096
alpha_c[8]	0.041779	4.049998	-6.799628	5.867400	1297.588475	1.000676
alpha_c[9]	-0.101276	2.371881	-4.027126	3.557981	554.458887	1.001785
alpha_c[10]	-0.206854	2.295808	-3.828741	3.446527	447.030524	1.001133
alpha_c[11]	0.276340	5.700333	-8.789997	9.354882	1898.253164	1.000677
alpha_c[12]	-0.634243	2.299146	-4.282144	2.974842	447.418059	1.001109
alpha_c[13]	-0.181725	5.807162	-9.099590	9.115609	1918.575787	0.999905
alpha_c[14]	0.290346	4.046932	-6.533361	6.179828	1290.857312	1.000625
alpha_c[15]	0.256918	2.374059	-3.738065	3.911657	557.908581	1.001656
alpha_c[16]	-0.639732	2.297209	-4.311422	2.984528	447.504145	1.001034
alpha_c[17]	-0.182213	3.245124	-5.635612	4.698237	811.658616	1.000977
alpha_c[18]	-0.633626	2.300197	-4.164071	3.109841	444.422404	1.001122
alpha_c[19]	0.033098	5.842382	-9.084964	9.442323	1983.164286	1.001918
alpha_c[20]	0.915345	2.294660	-2.594375	4.654440	444.848059	1.001045
alpha_c[21]	0.679118	2.371808	-3.249718	4.361766	554.694610	1.001768
alpha_c[22]	-0.885295	2.376394	-4.640897	3.000152	558.249572	1.001827
alpha_c[23]	0.141922	2.372421	-3.691759	3.937222	550.753852	1.001763
alpha_c[24]	0.253360	5.809715	-8.656647	9.504183	1936.843228	0.999912
alpha_c[25]	-0.800497	2.302991	-4.317002	2.948570	445.350663	1.001160
alpha_c[26]	0.070442	5.847059	-9.328448	9.170930	1984.910422	1.001872
alpha_c[27]	0.624014	4.476486	-6.325213	7.983405	1535.365067	1.000827
alpha_c[28]	-0.250690	5.191229	-9.351212	7.175612	1907.868209	0.999030
alpha_c[29]	-0.028963	4.469536	-6.691070	7.602648	1525.193226	1.000873
alpha_c[30]	-0.050877	3.246346	-5.450595	4.856264	815.563357	1.001015
alpha_c[31]	-0.254222	2.373687	-4.185421	3.412918	554.655633	1.001840
alpha_c[32]	0.241205	2.298255	-3.278584	4.014953	445.475815	1.001140
alpha_c[33]	0.103628	7.243771	-11.054058	11.743878	2241.631161	1.002949
alpha_c[34]	-0.501833	3.249136	-5.894413	4.438124	812.116588	1.000983
alpha_c[35]	-0.545980	3.258068	-6.071694	4.310493	814.088393	1.001077
alpha_c[36]	-0.248933	3.251855	-5.659408	4.669315	811.363734	1.001023
alpha_c[37]	0.352867	2.297679	-3.270450	3.993758	446.387224	1.001157
alpha_c[38]	0.003607	5.192583	-8.809873	7.745284	1900.821821	0.999010
alpha_c[39]	0.228948	2.300100	-3.401612	3.992358	445.807291	1.001211
alpha_c[40]	-0.031350	2.296017	-3.706045	3.565342	445.984750	1.001115
alpha_c[41]	-0.342952	3.247626	-5.966677	4.336488	810.706360	1.001020
alpha_c[42]	0.538346	2.374808	-3.265443	4.353185	553.233802	1.001741
alpha_c[43]	0.253050	2.372463	-3.459813	4.167420	554.347169	1.001767
alpha_c[44]	-0.230125	4.530264	-7.412365	6.863697	1412.749276	0.999910
alpha_c[45]	0.484974	2.372069	-3.439795	4.178841	555.646210	1.001770
alpha_c[46]	-0.519347	4.468183	-7.437415	6.864081	1530.461953	1.000891
alpha_c[47]	-0.184617	2.295639	-3.832515	3.450687	446.641411	1.001115
alpha_c[48]	0.087809	5.187164	-8.742705	7.777244	1903.259597	0.999020
alpha_c[49]	-0.856114	4.048944	-7.613929	4.982933	1292.502852	1.000537

	Mean	StdDev	lower 0.89	upper 0.89	n_eff	Rhat
alpha_c[50]	0.099500	2.296203	-3.505318	3.801629	445.413906	1.001141
alpha_c[51]	0.063683	2.297778	-3.694222	3.618509	444.236862	1.001137
alpha_c[52]	-0.016600	4.053057	-6.745072	5.934290	1294.630423	1.000663
alpha_c[53]	-0.166827	4.047203	-7.003189	5.602394	1298.832974	1.000608
alpha_c[54]	-0.312144	2.374881	-4.202905	3.417675	551.917252	1.001902
alpha_c[55]	0.429139	7.120868	-10.902938	11.602469	2680.402026	1.000902
alpha_c[56]	0.397628	2.298461	-3.232804	4.095586	446.186228	1.001108
alpha_c[57]	0.678611	2.375681	-3.183179	4.441004	552.472921	1.001720
alpha_c[58]	0.086992	2.373196	-3.570889	4.056486	555.770756	1.001731
alpha_c[59]	0.136656	5.694529	-9.111325	9.008139	1890.550946	1.000711
alpha_c[60]	0.207947	7.176807	-11.375254	11.324755	3110.349298	0.998463
alpha_c[61]	-0.901573	2.370966	-4.875266	2.700188	557.502184	1.001837
alpha_c[62]	-0.217731	3.248035	-5.633705	4.718966	811.588140	1.001105
alpha_c[63]	0.032273	7.123554	-11.829879	10.799049	2466.345257	0.999280
alpha_c[64]	0.043031	4.533366	-6.873333	7.402455	1411.057653	0.999905
alpha_c[65]	-0.316410	2.374098	-4.253650	3.354465	555.413811	1.001751
alpha_c[66]	-0.110257	2.297216	-3.645936	3.592441	446.742104	1.001138
alpha_c[67]	-0.689806	2.299406	-4.350598	2.915009	447.128195	1.001214
alpha_c[68]	-0.199468	3.248960	-5.471622	4.869576	813.687593	1.000944
alpha_c[69]	0.671979	4.528342	-6.543707	7.735890	1411.794813	0.999930
alpha_c[70]	-0.533531	4.531220	-7.331919	6.875648	1411.092397	0.999923
alpha_h[1]	-0.428338	4.167842	-7.179645	6.058170	1072.476214	1.000292
alpha_h[2]	-0.142395	3.727639	-6.816854	5.077703	972.075410	1.002307
alpha_h[3]	0.040142	3.753929	-6.133023	5.580322	830.622385	1.003403
alpha_h[4]	-0.457548	5.085619	-8.115758	8.262610	1472.163356	1.002866
alpha_h[5]	0.617398	4.819627	-6.948821	8.138337	1366.942240	1.000513
alpha_h[6]	0.156099	5.907751	-9.638052	9.325121	1687.445504	0.998767
alpha_h[7]	-0.017224	6.186389	-9.300604	10.275232	1989.229609	1.000241
alpha_h[8]	0.506651	5.988072	-7.945932	11.101157	2142.042205	0.999710
alpha_h[9]	-0.480123	5.734828	-8.933481	9.505306	1850.219829	0.998411
alpha_h[10]	0.538016	7.151562	-10.945446	12.284187	2893.949214	1.001839
alpha_h[11]	-0.369941	5.183292	-8.741951	7.489614	1547.325927	0.999065
alpha_h[12]	0.424340	7.200267	-10.445733	12.849755	2712.181446	1.000561
alpha_h[13]	-0.169622	7.211446	-12.122442	10.858815	2840.054879	0.998530
alpha_h[14]	-0.148872	7.076739	-12.016860	10.372658	2274.147610	0.999887
sigma	0.149222	0.007611	0.137128	0.161024	1248.616710	1.005426
betaGDP	0.091629	0.025236	0.051266	0.132341	2964.881301	0.998788
betaRAT	0.332689	0.050146	0.256342	0.415434	2526.719664	0.998920
betaCOS	0.202735	0.080363	0.074913	0.330147	2670.117578	0.999137
betaEDS	0.015714	0.022350	-0.017414	0.053592	2591.137762	0.998912
betaLEX	0.058944	0.024562	0.021865	0.099183	2620.912241	0.999099
betaDEM	-0.007764	0.024751	-0.045038	0.031789	3346.406152	0.999605

Dependent variable: Education E

	Mean	StdDev	lower 0.89	upper 0.89	n_eff	Rhat
alpha_g	0.170242	2.999368	-4.654221	4.818938	1473.573818	0.999879
alpha_c[1]	-0.268285	3.119559	-5.321520	4.365984	885.236974	1.003566
alpha_c[2]	-0.141964	2.175320	-3.327938	3.389766	435.493214	1.001933
alpha_c[3]	-0.183398	2.395163	-3.971882	3.384533	584.686168	1.001516
alpha_c[4]	0.140931	2.392237	-3.644397	3.750139	584.554855	1.001575
alpha_c[5]	0.249842	4.574684	-6.551480	7.842847	1632.406683	0.999410
alpha_c[6]	-0.114294	2.399214	-3.842864	3.607011	590.404533	1.001481
alpha_c[7]	0.068658	2.174901	-3.092153	3.649236	440.852095	1.002154
alpha_c[8]	-0.266331	4.126052	-7.520938	5.623370	1281.234541	1.002604
alpha_c[9]	0.112455	2.394977	-3.626865	3.720114	582.008882	1.001475
alpha_c[10]	0.393850	2.165754	-2.949924	3.839528	438.829974	1.001823
alpha_c[11]	-0.349944	5.734568	-9.709405	8.666439	2295.287861	0.998405
alpha_c[12]	0.621006	2.181289	-2.604309	4.221405	438.330360	1.001958
alpha_c[13]	0.270730	5.922283	-8.228667	10.196834	2610.552083	1.000091
alpha_c[14]	-0.728295	4.140981	-7.559648	5.627145	1285.877751	1.002678
alpha_c[15]	-0.369470	2.394386	-4.133694	3.203758	590.864972	1.001448
alpha_c[16]	0.139218	2.188943	-3.115492	3.723290	445.471236	1.001822
alpha_c[17]	0.883141	2.175710	-2.289634	4.494298	439.007739	1.001947
alpha_c[18]	0.267390	3.119091	-4.767862	4.903431	888.614592	1.003681
alpha_c[19]	0.839973	2.176257	-2.336012	4.423452	446.571115	1.001510
alpha_c[20]	-0.337139	5.981631	-10.889189	8.339552	2439.583263	0.998562
alpha_c[21]	-0.893748	2.173985	-4.369620	2.426600	444.932034	1.001691
alpha_c[22]	-0.453713	2.396875	-4.057760	3.281290	589.357793	1.001538
alpha_c[23]	1.741902	2.406495	-1.950167	5.567679	593.766300	1.001490
alpha_c[24]	-0.033552	2.396639	-3.798754	3.573825	584.408913	1.001561
alpha_c[25]	-0.397202	5.915431	-9.562159	8.932207	2607.972190	1.000218
alpha_c[26]	0.419036	2.186629	-2.639236	4.080319	435.724571	1.002015
alpha_c[27]	-0.018772	5.984710	-10.797691	8.519350	2431.129160	0.998574
alpha_c[28]	-0.416141	4.581164	-7.456906	7.036755	1633.806254	0.999508
alpha_c[29]	0.490905	4.899015	-7.878223	7.666179	1753.541441	1.001506
alpha_c[30]	0.764314	4.581112	-6.124035	8.384183	1639.623950	0.999507
alpha_c[31]	-0.723808	3.113478	-5.776610	3.911911	884.772528	1.003880
alpha_c[32]	0.704089	2.398083	-3.174166	4.219644	585.134139	1.001323
alpha_c[33]	-0.163693	2.173574	-3.447813	3.340590	439.823097	1.001668
alpha_c[34]	-0.144258	7.319644	-12.351112	10.662159	3067.034770	0.998859
alpha_c[35]	1.062616	3.120552	-3.889149	5.806670	900.184247	1.003607
alpha_c[36]	0.546910	3.122404	-4.349499	5.474814	889.928525	1.003794
alpha_c[37]	1.016934	3.115178	-4.205086	5.480163	880.457350	1.004029
alpha_c[38]	-0.818144	2.180459	-3.992003	2.763222	441.650552	1.001867
alpha_c[39]	-0.135665	4.890627	-8.317992	7.272384	1746.384119	1.001400
alpha_c[40]	0.423494	2.178070	-2.856529	3.959935	450.419458	1.001627
alpha_c[41]	0.177469	2.168234	-2.958829	3.852116	442.847197	1.001799
alpha_c[42]	-0.398531	3.109543	-5.597031	4.047171	885.921915	1.003783
alpha_c[43]	-0.621688	2.396712	-4.293553	3.073585	590.372161	1.001195
alpha_c[44]	-0.126949	2.399588	-3.930960	3.452137	588.609785	1.001344
alpha_c[45]	0.079432	4.555855	-7.842878	6.628079	1756.283583	1.001083
alpha_c[46]	-0.637254	2.397041	-4.547878	2.810660	588.494425	1.001456
alpha_c[47]	-0.185100	4.573840	-7.128993	7.385002	1636.587044	0.999508
alpha_c[48]	0.703008	2.177937	-2.457648	4.330328	439.356672	1.001891
alpha_c[49]	-0.075547	4.891953	-8.244502	7.241980	1763.026990	1.001549

	Mean	StdDev	lower 0.89	upper 0.89	n_eff	Rhat
alpha_c[50]	0.542141	4.135472	-6.547461	6.690628	1275.126877	1.002694
alpha_c[51]	-0.114878	2.167852	-3.375188	3.409096	438.543866	1.001993
alpha_c[52]	0.653979	2.178643	-2.694936	4.124263	447.886462	1.001797
alpha_c[53]	0.293246	4.128276	-6.409553	6.731162	1265.661415	1.002606
alpha_c[54]	-0.014406	4.129556	-7.385724	5.833324	1278.926888	1.002559
alpha_c[55]	0.878346	2.398314	-2.832872	4.607895	591.000011	1.001708
alpha_c[56]	0.084788	7.222923	-11.827368	11.119929	2905.520053	1.000296
alpha_c[57]	-0.304913	2.175867	-3.438396	3.294472	440.246740	1.001840
alpha_c[58]	-1.078957	2.394063	-4.804735	2.576483	592.563746	1.001363
alpha_c[59]	-0.569888	2.398367	-4.370691	2.990715	589.554665	1.001369
alpha_c[60]	0.355400	5.741265	-9.836352	8.596309	2297.942249	0.998416
alpha_c[61]	0.090080	7.078610	-11.631008	10.629572	2867.482951	0.999122
alpha_c[62]	1.233382	2.401124	-2.698708	4.741811	588.371668	1.001493
alpha_c[63]	-0.142093	3.116836	-5.047559	4.714593	896.400209	1.003920
alpha_c[64]	0.392814	7.240264	-11.291253	11.692772	3768.480114	0.998839
alpha_c[65]	-0.252477	4.547343	-7.927862	6.626706	1759.004763	1.001032
alpha_c[66]	0.562417	2.396345	-3.234266	4.119681	586.806989	1.001437
alpha_c[67]	-0.500559	2.175362	-3.694630	3.039053	438.089339	1.002024
alpha_c[68]	0.532956	2.180643	-2.659785	4.097446	435.509338	1.001994
alpha_c[69]	-0.478528	3.114540	-5.597333	4.115873	891.894007	1.003754
alpha_c[70]	0.222963	4.556969	-7.598980	6.888810	1754.593085	1.001101
alpha_c[71]	0.423908	4.556121	-7.329357	7.224153	1754.973499	1.001091
alpha_h[1]	1.004251	4.140247	-5.460683	7.493185	1003.654835	1.003020
alpha_h[2]	-0.367718	3.479129	-5.937485	5.164745	931.850770	1.000771
alpha_h[3]	-0.780554	3.648573	-6.852340	4.757336	928.060179	1.001360
alpha_h[4]	0.296452	5.155299	-8.116987	8.242496	1644.929377	0.999793
alpha_h[5]	-0.192976	4.805750	-7.865836	7.591853	1368.026370	1.002112
alpha_h[6]	0.091879	6.025133	-10.143579	9.001631	2141.269397	0.998743
alpha_h[7]	0.007650	6.116896	-9.152910	10.326963	2308.403622	1.000693
alpha_h[8]	-0.409351	6.248058	-10.427021	9.612432	2028.182466	0.998621
alpha_h[9]	0.572728	5.316335	-7.490832	8.988037	1725.651641	1.002359
alpha_h[10]	-0.199251	7.424183	-12.773670	10.944740	2990.376375	0.998999
alpha_h[11]	0.199432	4.969569	-7.561177	8.224381	1736.900430	1.001852
alpha_h[12]	0.010597	7.184463	-10.747703	12.023921	2904.659125	1.000493
alpha_h[13]	-0.423267	7.231625	-12.012625	10.500840	2929.361976	0.999421
alpha_h[14]	0.349374	7.237477	-10.972266	11.607781	3710.402877	0.998594
sigma	0.358030	0.014586	0.335205	0.381615	2169.669341	0.999248
betaGDP	-0.088911	0.044803	-0.155773	-0.015266	2686.575562	0.998893
betaRAT	0.746903	0.104198	0.571107	0.904614	3507.424839	0.999558
betaCOS	0.189497	0.138266	-0.041757	0.402002	4320.997999	0.999640
betaEDS	0.079094	0.051625	-0.003154	0.159060	3233.614876	0.998991
betaLEX	0.521276	0.046782	0.445456	0.594852	3955.503740	0.998369
betaDEM	0.090984	0.040746	0.023192	0.150919	4264.400216	0.998935

Dependent variable: Life Expectancy L

	Mean	StdDev	lower 0.89	upper 0.89	n_eff	Rhat
alpha_g	0.125895	3.030937	-5.180972	4.544522	1800.645426	0.999519
alpha_c[1]	0.305471	3.248076	-4.844644	5.514024	1061.296820	1.005288
alpha_c[2]	0.052194	2.071655	-3.286324	3.220262	376.886170	1.012110
alpha_c[3]	-0.001325	2.394007	-4.010705	3.583844	496.404157	1.008302
alpha_c[4]	0.109037	2.394500	-3.870409	3.681872	497.738027	1.008425
alpha_c[5]	0.021139	4.784738	-7.365934	7.637583	1892.209808	0.998548
alpha_c[6]	-0.044819	2.388692	-4.062907	3.530197	494.466709	1.008347
alpha_c[7]	0.059331	2.070510	-3.256627	3.216668	382.340486	1.011811
alpha_c[8]	0.059255	4.253146	-6.527029	6.739813	1741.353616	1.001431
alpha_c[9]	0.117696	2.391849	-3.901817	3.679014	495.268679	1.008518
alpha_c[10]	0.297997	2.067461	-3.025324	3.440085	385.247000	1.011943
alpha_c[11]	0.179032	5.857409	-9.391761	9.159142	3117.078034	0.999728
alpha_c[12]	0.162144	2.071140	-3.220669	3.287802	380.343360	1.011940
alpha_c[13]	0.029949	6.023416	-9.530025	9.463695	3560.515555	0.998743
alpha_c[14]	0.044644	4.258965	-6.703748	6.610814	1751.813331	1.001434
alpha_c[15]	-0.067021	2.393147	-4.256447	3.297978	493.382334	1.008476
alpha_c[16]	0.137331	2.066556	-3.056694	3.454514	382.374291	1.011760
alpha_c[17]	0.337633	2.068229	-2.993979	3.490040	384.704807	1.011856
alpha_c[18]	-0.083351	3.242971	-5.297703	5.089131	1056.670178	1.005392
alpha_c[19]	-0.009479	2.071955	-3.133072	3.381958	378.821947	1.011951
alpha_c[20]	0.092073	5.648709	-8.243403	10.150892	3020.103006	0.999631
alpha_c[21]	0.009880	2.061210	-3.304717	3.160684	384.381282	1.012107
alpha_c[22]	-0.132119	2.395796	-4.326456	3.225167	495.416226	1.008470
alpha_c[23]	-0.458356	2.412938	-4.669245	2.952829	496.271221	1.009248
alpha_c[24]	-0.013334	2.393269	-4.152211	3.407397	494.066400	1.008552
alpha_c[25]	0.076946	6.023905	-9.498364	9.464440	3566.351646	0.998715
alpha_c[26]	0.247971	2.082816	-3.162940	3.361109	379.902778	1.011453
alpha_c[27]	-0.066736	5.647973	-8.964233	9.488184	3044.955499	0.999568
alpha_c[28]	-0.340599	4.795600	-7.948628	7.012418	1898.329583	0.998575
alpha_c[29]	0.023281	4.873498	-7.180570	8.251742	2237.811780	0.999359
alpha_c[30]	0.475756	4.785736	-6.816995	8.191981	1905.028193	0.998570
alpha_c[31]	-0.124222	3.252212	-5.360612	5.080636	1062.342544	1.005064
alpha_c[32]	0.216734	2.392857	-3.810117	3.765344	492.860670	1.008743
alpha_c[33]	0.228329	2.067407	-3.099349	3.380487	384.624370	1.011577
alpha_c[34]	-0.010419	7.006489	-10.941158	11.685300	3509.416045	0.999867
alpha_c[35]	0.213519	3.249521	-5.064121	5.380373	1061.004394	1.005399
alpha_c[36]	-0.346736	3.257163	-5.470205	5.015495	1067.552743	1.005072
alpha_c[37]	-0.014669	3.253938	-5.362730	5.083974	1062.890258	1.005117
alpha_c[38]	-0.082797	2.066035	-3.493560	2.987455	381.491210	1.011564
alpha_c[39]	0.068945	4.880766	-7.089834	8.310056	2239.444449	0.999336
alpha_c[40]	-1.036759	2.075175	-4.445687	2.094667	381.377672	1.011638
alpha_c[41]	0.216111	2.069265	-3.122665	3.394040	385.301290	1.011096
alpha_c[42]	0.116269	3.253517	-5.133719	5.288172	1061.500720	1.005152
alpha_c[43]	-0.003090	2.393548	-4.303742	3.299907	496.117506	1.008335
alpha_c[44]	-0.084227	2.390060	-4.239167	3.295499	493.037810	1.008535
alpha_c[45]	-0.297620	4.486984	-7.718446	6.578448	2033.286407	0.998650
alpha_c[46]	0.022998	2.388086	-4.113757	3.450077	490.752300	1.008388
alpha_c[47]	0.122072	4.784469	-7.581199	7.457727	1901.743897	0.998565
alpha_c[48]	0.110650	2.064295	-3.144931	3.336430	371.102664	1.012355
alpha_c[49]	-0.310246	4.883420	-7.357456	8.100056	2244.392719	0.999445

	Mean	StdDev	lower 0.89	upper 0.89	n_eff	Rhat
alpha_c[50]	0.281663	4.257180	-6.620897	6.676809	1746.128878	1.001415
alpha_c[51]	0.296280	2.071175	-3.001157	3.515291	381.846454	1.011600
alpha_c[52]	0.006065	2.071780	-3.329172	3.115990	372.302455	1.011650
alpha_c[53]	-0.608988	4.255674	-7.154428	6.168496	1746.049200	1.001414
alpha_c[54]	0.074433	4.259133	-6.555083	6.759482	1747.997529	1.001556
alpha_c[55]	-0.908492	2.395087	-5.027132	2.580442	495.642255	1.008705
alpha_c[56]	0.065102	7.310539	-12.758478	10.594756	3018.194853	0.999162
alpha_c[57]	0.278141	2.066665	-3.144377	3.380302	385.755092	1.011837
alpha_c[58]	-0.010402	2.392996	-4.113778	3.390141	493.110518	1.008258
alpha_c[59]	0.095704	2.390839	-3.894358	3.675142	497.894128	1.008226
alpha_c[60]	0.080777	5.851974	-9.623495	8.902786	3106.213405	0.999772
alpha_c[61]	-0.007962	7.041890	-10.529433	11.488347	2981.167334	1.000216
alpha_c[62]	-0.194302	2.397088	-4.235929	3.357925	495.958304	1.008542
alpha_c[63]	0.390967	3.253644	-5.027594	5.450160	1060.562182	1.005273
alpha_c[64]	-0.046918	7.303932	-12.080057	11.068886	3076.903064	1.000404
alpha_c[65]	-0.103781	4.489068	-7.547873	6.639039	2031.187007	0.998596
alpha_c[66]	-0.056783	2.392243	-4.271031	3.280461	497.631104	1.008593
alpha_c[67]	0.109616	2.071446	-3.243834	3.234369	378.333480	1.011783
alpha_c[68]	0.089319	2.071392	-3.350100	3.164699	377.964614	1.012028
alpha_c[69]	-0.311128	3.252885	-5.588656	4.795452	1057.100196	1.005270
alpha_c[70]	-0.368359	4.489753	-7.985180	6.246863	2035.270204	0.998627
alpha_c[71]	-0.305595	4.488712	-7.576204	6.696378	2038.532049	0.998623
alpha_h[1]	0.039066	4.266517	-6.959140	6.602386	1150.369058	1.002109
alpha_h[2]	-0.058405	3.657842	-6.060212	5.530859	1008.268016	1.003666
alpha_h[3]	-0.128761	3.755584	-6.514174	5.421546	874.016613	1.002757
alpha_h[4]	-0.365045	5.255214	-8.431128	8.366740	1818.320024	0.998944
alpha_h[5]	-0.279554	4.984262	-8.754215	6.837985	1712.663783	1.000043
alpha_h[6]	-0.003039	6.039621	-9.449481	9.238541	2790.489742	1.001000
alpha_h[7]	0.060962	6.205754	-9.527855	10.307259	2916.175981	0.998769
alpha_h[8]	-0.146924	5.889419	-9.011059	10.106136	2741.615854	1.000393
alpha_h[9]	-0.129118	5.388275	-8.913193	8.148640	1799.178986	0.999735
alpha_h[10]	0.162430	7.002590	-10.116365	12.399061	3442.088102	0.999260
alpha_h[11]	-0.991641	4.924363	-7.821831	8.227987	1934.152682	0.999917
alpha_h[12]	-0.040239	7.279551	-12.090257	11.319486	3303.669081	0.999320
alpha_h[13]	-0.055867	7.102711	-11.647722	11.178163	3447.129421	1.000743
alpha_h[14]	-0.110988	7.274419	-11.447618	11.604905	2949.245161	0.999842
sigma	0.279265	0.011107	0.262500	0.296749	1771.544409	1.001355
betaGDP	0.053735	0.036244	-0.000448	0.113623	3573.945652	0.998535
betaRAT	0.150559	0.080348	0.026954	0.279061	3464.510231	0.999524
betaCOS	-0.070332	0.103362	-0.243148	0.091818	3450.042303	0.999947
betaEDS	0.028957	0.041202	-0.035984	0.093749	2596.965784	1.000164
betaLEX	0.659214	0.035841	0.596530	0.710723	2696.159906	0.998469
betaDEM	0.068097	0.031906	0.012741	0.112630	3049.049872	0.999480

Dependent variable: GDP per Capita GDP

	Mean	StdDev	lower 0.89	upper 0.89	n_eff	Rhat
alpha_g	0.047264	3.069969	-4.718298	5.113782	1691.636306	1.001172
alpha_c[1]	-0.468587	3.184289	-5.778984	4.207607	1176.399431	0.999716
alpha_c[2]	-0.188747	2.354234	-3.888147	3.634032	536.719848	1.004794
alpha_c[3]	0.008278	2.326605	-3.794443	3.564419	595.851750	1.006308
alpha_c[4]	0.199552	2.331200	-3.505066	3.915951	591.441083	1.006416
alpha_c[5]	0.085472	4.377392	-6.428705	7.401366	1598.001889	0.999352
alpha_c[6]	-0.143147	2.320612	-4.108192	3.270084	593.271711	1.005743
alpha_c[7]	-0.206360	2.357579	-3.791856	3.654188	536.115086	1.005037
alpha_c[8]	-0.092520	4.036586	-6.389717	6.359974	1397.883016	1.000839
alpha_c[9]	0.421100	2.322443	-3.455082	3.929560	595.075960	1.006074
alpha_c[10]	-0.052515	2.364561	-3.733463	3.734497	538.025546	1.004509
alpha_c[11]	-0.756460	5.956395	-9.886800	9.184033	2463.475016	0.998591
alpha_c[12]	0.141881	2.361947	-3.646782	3.867882	538.315262	1.004992
alpha_c[13]	0.161041	6.092246	-8.970177	10.401433	2286.686271	0.998819
alpha_c[14]	-0.239326	4.035681	-6.806549	5.993324	1403.014226	1.000843
alpha_c[15]	-0.183567	2.327083	-4.179142	3.209985	582.174727	1.006067
alpha_c[16]	-0.178901	2.361419	-4.007549	3.538750	536.379695	1.004490
alpha_c[17]	0.263336	2.360349	-3.560422	3.961874	530.683036	1.005194
alpha_c[18]	-0.423492	3.181240	-5.749809	4.215726	1179.137886	0.999757
alpha_c[19]	0.351248	2.362329	-3.285941	4.283231	535.756282	1.005060
alpha_c[20]	-0.187128	5.784189	-9.304913	8.884309	2004.582749	0.999429
alpha_c[21]	-0.735287	2.354445	-4.576386	2.908842	539.227458	1.004958
alpha_c[22]	-0.332606	2.329860	-4.012165	3.368002	586.820168	1.006003
alpha_c[23]	0.560923	2.334079	-3.130867	4.302634	600.426537	1.005537
alpha_c[24]	-0.025405	2.326329	-3.942522	3.467504	591.568928	1.005700
alpha_c[25]	-0.552841	6.085896	-9.653351	9.749937	2290.448717	0.998855
alpha_c[26]	0.094438	2.364228	-3.514456	3.956822	532.600218	1.004973
alpha_c[27]	-0.324628	5.792526	-9.641850	8.527283	2006.607363	0.999427
alpha_c[28]	-0.732276	4.377062	-7.545747	6.231039	1599.651481	0.999349
alpha_c[29]	0.276863	5.140453	-7.567836	8.699877	2077.519062	0.998674
alpha_c[30]	0.230988	4.380742	-6.666639	7.125087	1592.022179	0.999401
alpha_c[31]	-0.423644	3.183173	-5.951880	4.005736	1173.470300	0.999723
alpha_c[32]	0.811971	2.323053	-2.822756	4.580934	600.221473	1.005554
alpha_c[33]	0.136212	2.356354	-3.519693	3.987885	534.853081	1.005270
alpha_c[34]	-0.531728	7.326484	-12.548075	10.630803	3654.828886	1.000842
alpha_c[35]	0.249682	3.185767	-5.097649	4.871880	1183.218064	0.999803
alpha_c[36]	1.494574	3.206556	-4.056802	5.973783	1185.509394	0.999738
alpha_c[37]	0.392946	3.182967	-4.887876	4.949547	1177.602878	0.999673
alpha_c[38]	0.662713	2.356324	-3.117405	4.376495	542.301093	1.004884
alpha_c[39]	0.384267	5.147673	-7.155440	9.350110	2084.303833	0.998725
alpha_c[40]	-0.641490	2.356769	-4.274919	3.253548	538.317021	1.004909
alpha_c[41]	-0.135094	2.358464	-3.901102	3.558909	533.600306	1.004865
alpha_c[42]	-0.067913	3.181057	-5.410572	4.567898	1172.118462	0.999888
alpha_c[43]	-0.260072	2.331362	-4.212999	3.232747	608.332770	1.005980
alpha_c[44]	-0.541270	2.333466	-4.449011	2.995150	590.812090	1.006160
alpha_c[45]	0.394108	4.601931	-6.797657	7.831962	1695.218534	0.998680
alpha_c[46]	0.497226	2.327839	-3.336438	4.044552	602.905155	1.005987
alpha_c[47]	0.215238	4.384590	-6.294747	7.454467	1597.070331	0.999399
alpha_c[48]	-0.377534	2.353803	-4.040712	3.392550	538.368744	1.004709
alpha_c[49]	-0.493678	5.144632	-8.355715	7.962544	2085.790923	0.998749

	Mean	StdDev	lower 0.89	upper 0.89	n_eff	Rhat
alpha_c[50]	0.666613	4.047565	-5.597764	7.128203	1409.454012	1.000892
alpha_c[51]	0.020293	2.359003	-4.089450	3.459318	533.429898	1.005142
alpha_c[52]	-0.106493	2.360888	-3.923447	3.578848	534.140764	1.005368
alpha_c[53]	-0.193535	4.032889	-7.064308	5.740723	1394.424546	1.000777
alpha_c[54]	-0.173111	4.039528	-6.293470	6.451191	1401.741403	1.000751
alpha_c[55]	0.104979	2.326959	-3.572025	3.852676	592.289101	1.005742
alpha_c[56]	-0.234329	7.036893	-11.675184	11.098854	2736.334137	0.998757
alpha_c[57]	-0.314682	2.354599	-4.048884	3.430887	541.374896	1.004872
alpha_c[58]	-0.628591	2.327725	-4.213648	3.171527	591.945554	1.006112
alpha_c[59]	0.485882	2.324019	-3.389937	4.020584	597.783730	1.006048
alpha_c[60]	0.423513	5.944700	-9.053328	10.028723	2427.858317	0.998638
alpha_c[61]	0.007962	7.484464	-11.014112	12.420344	2604.180046	1.000154
alpha_c[62]	0.881978	2.331478	-2.721641	4.735571	593.257082	1.005705
alpha_c[63]	-0.264594	3.182531	-5.464887	4.500451	1174.653948	0.999804
alpha_c[64]	0.151345	7.003121	-11.494762	10.385659	2570.621777	0.999785
alpha_c[65]	-0.197919	4.603252	-7.343795	7.201345	1702.780075	0.998644
alpha_c[66]	0.795542	2.324821	-2.814356	4.587722	594.541370	1.005775
alpha_c[67]	-0.602918	2.357220	-4.337670	3.078688	540.116179	1.004718
alpha_c[68]	0.549010	2.362433	-3.086203	4.481713	538.534923	1.005091
alpha_c[69]	-0.462982	3.187311	-5.746987	4.179153	1169.719433	0.999962
alpha_c[70]	-0.630426	4.601355	-7.852009	6.697061	1702.267515	0.998678
alpha_c[71]	0.204106	4.601256	-6.933247	7.696618	1694.044650	0.998699
alpha_h[1]	0.602876	4.145615	-6.115974	7.065174	1371.958063	0.999750
alpha_h[2]	0.045083	3.753473	-6.251630	5.538508	1054.863729	1.003450
alpha_h[3]	-0.222441	3.648636	-6.322903	5.403789	1035.803408	1.002251
alpha_h[4]	0.043251	4.965716	-7.978508	7.508974	1655.794580	0.999536
alpha_h[5]	-0.715213	4.661589	-8.434941	6.298841	1583.541426	1.000140
alpha_h[6]	-0.145229	6.160429	-10.029432	9.325519	2355.172345	0.999381
alpha_h[7]	0.262909	6.318918	-10.401559	9.671408	2069.711115	0.999811
alpha_h[8]	-0.219370	6.008143	-9.572669	9.423932	2212.457757	0.999418
alpha_h[9]	0.233507	5.424514	-8.457777	8.348307	2044.326596	0.998457
alpha_h[10]	0.020928	7.414194	-11.249038	12.607331	3644.707464	0.999394
alpha_h[11]	-0.045461	5.299858	-8.325360	8.217777	1656.413506	0.998267
alpha_h[12]	-0.155827	7.086449	-12.308441	10.628207	2705.156038	0.999425
alpha_h[13]	-0.026101	7.562645	-12.161957	11.940327	2688.919593	0.999176
alpha_h[14]	0.311441	7.150149	-9.933149	12.561656	2604.199307	0.999520
sigma	0.349078	0.014515	0.326929	0.373276	1661.949538	1.001559
betaGDP	0.239126	0.043242	0.162499	0.300380	2832.357583	0.999639
betaRAT	0.664340	0.096844	0.510084	0.817866	2781.885581	1.001002
betaCOS	0.146176	0.127180	-0.053615	0.354937	3025.467517	0.999494
betaEDS	0.273904	0.053251	0.194079	0.356750	2647.879451	0.998924
betaLEX	-0.132044	0.043650	-0.192371	-0.050995	2924.059910	0.998413
betaDEM	-0.018211	0.039467	-0.083531	0.040848	2868.631650	1.000262

Dependent variable: Democracy D

	Mean	StdDev	lower 0.89	upper 0.89	n_eff	Rhat
alpha_g	0.178794	3.164184	-4.670125	5.633332	2070.223050	1.001148
alpha_c[1]	0.325518	3.111617	-4.453080	5.351925	1059.470079	1.001517
alpha_c[2]	-1.187204	2.267527	-4.751941	2.517037	581.968820	1.004049
alpha_c[3]	0.069590	2.345830	-3.433251	3.934641	573.766522	1.002216
alpha_c[4]	0.169563	2.353225	-3.645383	3.717218	575.443302	1.001964
alpha_c[5]	-0.045770	4.506987	-6.964411	7.267826	2111.955146	0.999225
alpha_c[6]	0.304460	2.347700	-3.323106	4.129515	577.295350	1.002223
alpha_c[7]	-0.659813	2.270489	-4.162739	3.096357	584.816022	1.003529
alpha_c[8]	-0.319060	4.139204	-7.777036	5.870952	1540.715356	1.002336
alpha_c[9]	-0.029541	2.335090	-3.723247	3.683250	577.749118	1.002160
alpha_c[10]	-0.139171	2.259843	-3.604852	3.627999	580.367454	1.003192
alpha_c[11]	-0.596917	5.416552	-8.769268	8.396447	2797.848949	0.999382
alpha_c[12]	0.182618	2.261346	-3.701473	3.577504	580.066822	1.003863
alpha_c[13]	0.201479	5.885309	-8.934698	9.614681	3023.626466	0.999786
alpha_c[14]	0.718570	4.139885	-6.544314	6.905309	1522.938992	1.002198
alpha_c[15]	-0.443592	2.364377	-4.254122	3.194959	578.192930	1.002110
alpha_c[16]	0.277838	2.282052	-3.345215	3.955349	597.429026	1.004116
alpha_c[17]	0.489712	2.265812	-2.943262	4.299839	578.814322	1.003992
alpha_c[18]	-0.485241	3.103254	-5.483730	4.417439	1046.263106	1.001733
alpha_c[19]	0.489260	2.266404	-3.397758	3.780432	582.676733	1.003423
alpha_c[20]	0.247839	5.815801	-9.606335	8.734143	2723.605355	0.999729
alpha_c[21]	0.326787	2.266769	-3.045010	4.190255	579.439548	1.003903
alpha_c[22]	0.243599	2.356733	-3.700999	3.736706	574.906211	1.002090
alpha_c[23]	0.049106	2.375953	-3.770990	3.777362	583.221678	1.002067
alpha_c[24]	0.199927	2.347130	-3.477709	3.904782	571.277981	1.002425
alpha_c[25]	-0.268692	5.875708	-9.503706	8.911136	2956.455195	0.999782
alpha_c[26]	-0.391916	2.267470	-4.055916	3.139921	584.673861	1.003805
alpha_c[27]	-0.506711	5.805582	-9.395743	8.754616	2713.239079	0.999838
alpha_c[28]	1.925113	4.524966	-5.337722	8.964155	2098.941045	0.999191
alpha_c[29]	-0.546753	5.230448	-8.773942	8.154665	2283.330921	1.000562
alpha_c[30]	-1.170027	4.506246	-7.953199	6.318876	2102.077800	0.999304
alpha_c[31]	-0.379745	3.103913	-5.446594	4.360666	1049.469148	1.001753
alpha_c[32]	-0.065429	2.348312	-3.725180	3.689791	574.564134	1.002094
alpha_c[33]	0.659977	2.258581	-3.199702	3.997141	576.753357	1.003508
alpha_c[34]	0.469227	7.390432	-10.902768	13.032762	3955.987300	0.998415
alpha_c[35]	-0.344667	3.099662	-5.271492	4.535670	1020.344506	1.001543
alpha_c[36]	0.170278	3.134507	-4.805377	4.986132	1088.692931	1.000541
alpha_c[37]	0.153166	3.105488	-4.840776	4.942140	1024.378956	1.001334
alpha_c[38]	0.502068	2.271016	-3.281107	3.942404	582.614458	1.004191
alpha_c[39]	0.405322	5.242261	-8.483390	8.350498	2307.203174	1.000485
alpha_c[40]	1.137279	2.279769	-2.654352	4.645942	591.544064	1.003033
alpha_c[41]	-0.574998	2.266502	-4.195044	3.053087	576.860551	1.004146
alpha_c[42]	-0.972076	3.096143	-5.738232	3.920344	1083.929084	1.001610
alpha_c[43]	-0.132223	2.341819	-4.087920	3.376450	573.662303	1.002174
alpha_c[44]	-0.189236	2.347822	-3.877542	3.543054	576.290787	1.002404
alpha_c[45]	0.387737	4.565120	-6.533781	7.962639	2489.436275	0.999191
alpha_c[46]	-0.157622	2.347912	-3.925999	3.465363	574.318480	1.002233
alpha_c[47]	-0.673168	4.522130	-8.291975	6.050747	2093.693489	0.999135
alpha_c[48]	0.252447	2.260221	-3.253543	3.967817	577.328704	1.004080
alpha_c[49]	0.033671	5.238203	-8.229596	8.716639	2300.131665	1.000436

	Mean	StdDev	lower 0.89	upper 0.89	n_eff	Rhat
alpha_c[50]	-0.829052	4.144819	-7.940073	5.620608	1536.652491	1.002403
alpha_c[51]	-0.096879	2.264186	-3.745054	3.538670	577.189735	1.004132
alpha_c[52]	-0.312420	2.272712	-3.968908	3.283785	588.180974	1.003861
alpha_c[53]	0.273553	4.148800	-6.821680	6.701333	1497.797551	1.001678
alpha_c[54]	-0.835171	4.133658	-7.854170	5.681087	1545.452355	1.001996
alpha_c[55]	0.688084	2.349858	-3.202278	4.228815	579.954400	1.001507
alpha_c[56]	0.288382	7.397054	-11.592397	11.732723	3453.770971	0.998947
alpha_c[57]	-0.910349	2.269426	-4.434802	2.782057	569.293489	1.003867
alpha_c[58]	-0.263443	2.361329	-3.845631	3.646470	580.004358	1.001754
alpha_c[59]	-0.049269	2.344117	-3.765257	3.599954	576.231729	1.002315
alpha_c[60]	0.161881	5.423073	-7.771484	9.293992	2792.871286	0.999446
alpha_c[61]	0.353848	7.376769	-11.114635	11.904733	3031.906365	0.998971
alpha_c[62]	0.187196	2.356600	-3.717045	3.740101	579.015802	1.002235
alpha_c[63]	-0.491452	3.095476	-5.650588	4.006218	1050.318034	1.001388
alpha_c[64]	0.894634	7.140650	-10.555158	12.439154	2814.997677	0.999183
alpha_c[65]	-0.575633	4.565571	-7.253084	7.191094	2488.761048	0.999236
alpha_c[66]	0.271350	2.342290	-3.717474	3.789707	571.112943	1.002305
alpha_c[67]	-0.679060	2.273813	-4.146790	3.087989	584.194289	1.003685
alpha_c[68]	0.351970	2.267226	-3.200173	3.982018	590.778918	1.003817
alpha_c[69]	0.292682	3.098257	-4.431687	5.346768	1042.017744	1.001454
alpha_c[70]	0.830619	4.576525	-6.139144	8.316154	2498.996969	0.999132
alpha_c[71]	-1.064126	4.554653	-7.712665	6.744496	2487.955064	0.999301
alpha_h[1]	-0.520000	4.069700	-7.166758	5.739768	1451.962571	1.003120
alpha_h[2]	-0.319857	3.680137	-5.806606	5.932275	1250.385940	1.001242
alpha_h[3]	-0.027358	3.735799	-6.249581	5.581416	1125.375271	1.003328
alpha_h[4]	0.114473	5.178119	-7.531947	9.127361	1929.625357	0.999329
alpha_h[5]	-0.079911	4.866055	-8.131986	7.342959	1680.427386	1.001140
alpha_h[6]	-0.586410	5.891324	-9.725693	9.106310	2723.055791	0.999284
alpha_h[7]	0.007848	6.194975	-10.531148	9.122022	2963.468845	1.000883
alpha_h[8]	-0.093798	6.052951	-9.095200	10.091709	2785.967161	1.001058
alpha_h[9]	-0.051686	5.693382	-9.139652	9.234151	2204.432573	1.001316
alpha_h[10]	0.353029	7.229968	-10.798075	11.933814	3715.245536	0.998353
alpha_h[11]	0.078939	5.129267	-7.878055	8.243065	2413.737421	1.000177
alpha_h[12]	0.119973	7.544529	-11.958238	12.005685	3592.164832	0.998408
alpha_h[13]	0.152885	7.348216	-11.170492	11.986760	2896.425550	0.999466
alpha_h[14]	0.779821	7.167107	-10.520025	13.071009	3007.883800	0.998395
sigma	0.520694	0.023536	0.482840	0.556300	1381.053657	1.000306
betaGDP	-0.247970	0.080653	-0.369558	-0.115541	2759.149074	1.000092
betaRAT	-0.199936	0.159612	-0.450418	0.061445	3246.763691	0.999705
betaCOS	0.718192	0.221547	0.353689	1.059223	3001.563884	1.000314
betaEDS	0.145302	0.079352	0.017099	0.268704	2579.864290	0.999693
betaLEX	0.398852	0.077726	0.277613	0.526806	2914.288139	0.999832
betaDEM	-0.114039	0.076337	-0.230019	0.014435	2657.050207	0.999874
